# Core Mechanisms in Action Control: Binding and Retrieval

**DOI:** 10.5334/joc.253

**Published:** 2023-01-09

**Authors:** Andrea Kiesel, Lisa R. Fournier, Carina G. Giesen, Susanne Mayr, Christian Frings

**Affiliations:** 1 University of Freiburg, DE; 2 Washington State University, US; 3 Friedrich Schiller University Jena, DE; 4 University of Passau, DE; 5 Trier University, DE

**Keywords:** Action, Action and perception, Event cognition, Cognitive Control

## Abstract

This special collection focuses on action control and its two postulated core processes, namely feature binding and retrieval. Action control is an important topic as humans interact with their environment by means of goal-directed behavior, i.e. by means of actions. Cognitive processes were developed and shaped to enhance preparation, execution, and regulation of action. Therefore, it is the current consensus that cognition serves action.

To date, research on human action control is comprised mainly of an abundance of paradigm-specific results and models. To gain a better understanding of action control, an integrative framework was proposed (the BRAC framework – for Binding and Retrieval in Action Control, Frings et al., 2020) that can explain a wide range of findings across different experimental paradigms by assuming two core processes as key functions in action control: feature binding and feature retrieval. In this special collection, 20 articles present and discuss different types of sequential paradigms in terms of this integrative account. This editorial explains the major assumptions of the BRAC framework and provides an integrative overview of the articles that are included in this special collection.

Action control is arguably one of the most fundamental topics in cognition because humans interact with their environment by means of goal-directed behavior; that is, by means of actions. Cognitive processes were developed and shaped to enhance preparation, execution, and regulation of action. Therefore, cognition serves action (e.g., Allport, 1987). To date, research on human action control primarily comprises paradigm-specific experimental approaches and models. To gain a better understanding of action control across paradigm-specific results, Frings et al. (2020) proposed the Binding and Retrieval in Action Control (BRAC) framework. The BRAC framework assumes that *feature binding* and *feature retrieval* are two core processes in action control. Evaluating contributions of these two core processes in action control across a wide range of experimental paradigms will allow us to integrate these different paradigm-specific results to better understand action control.

Many experimental paradigms that analyze action control (e.g., task switching, negative priming, stimulus-response binding tasks, action planning tasks, or conflict tasks measuring cognitive control) use a sequential logic. For example, in task switching, performance in a current trial is analyzed by comparing whether the task on the previous trial repeats or switches (for overviews see Kiesel, et al., 2010; Koch, et al., 2018; Vandierendonck, et al., 2010). In negative priming, responding to a target in the current trial is analyzed by comparing whether the target (or features of the target) was a distractor or not in the previous trial (for overviews, see Frings et al., 2015; Mayr & Buchner, 2007). In stimulus-response binding tasks, performance is analyzed when responding to a specific target stimulus depending on whether the required response repeats or switches between two presentations of this specific target stimulus (item-specific repetition or switches of responses; e.g., Henson et al., 2014, Pfeuffer et al., 2017). In action planning tasks, performance to a probe event is analyzed by comparing conditions in which the probe event contains features that partly overlap, do not overlap, and/or completely overlap with a prime event retained in short-term or working memory (Stoet & Hommel, 1999; Wiediger & Fournier, 2008). Finally, in conflict tasks measuring cognitive control (e.g., in Stroop, Flanker, Simon or other types of conflict tasks), the congruency effect in the current trial is analyzed with regards to the congruency level of the previous trial (e.g., Dignath, et al., 2021; Gratton, et al., 1992; for overviews/theoretical accounts see Botvinick, et al., 2001; Dignath et al., 2020). Frings and colleagues (2020) argue that the empirical pattern of results obtained in these sequential paradigms can be accounted for by the two core mechanisms of feature binding and retrieval. In one event, the stimulus, response, and effect features are integrated/bound together, and in a succeeding event, these features are retrieved (although the definition of an event is somewhat different across paradigms). This novel framework of binding and retrieval allows different sequential experimental effects to be discussed conjointly, and it enables the assessment of whether processes of feature binding and retrieval can be independently modulated by top-down and bottom-up processes.

The 20 articles in this special collection present and discuss data from different sequential paradigms regarding a possible impact of binding and/or retrieval processes in action control. In the theoretical paper by Hommel (2022), the Theory of Event Coding (Hommel et al., 2001) is reconsidered and the concepts of object and event files are explained. Then, Hommel argues that it is theoretically important to distinguish binding and retrieval processes as suggested by the BRAC framework (Frings et al., 2020). Although a clear process distinction is empirically challenging, there is accumulating evidence that binding and retrieval processes differ in how they are influenced by other variables. Stimuli and actions seem to bind together spontaneously, thus binding can be conceptualized as a more automatic process, whereas retrieval is influenced by different modulators related to attention. In two experiments, Schmalbrock and colleagues (2022) provide further evidence for this conclusion. They manipulated perceptual grouping of distractors in a distractor-response binding paradigm and found that perceptual grouping did not influence binding mechanisms but modulated retrieval. These findings support the assumption that retrieval, but not binding, is influenced by processes modulated by attention. Consistent with this conclusion, Selimi and colleagues (2022) showed grouping of action effects may not affect response-response binding. They investigated whether visual after-effects of responses further binding and retrieval when these visual effects were grouped together – comparable to previous findings concerning influences of stimulus grouping effects. However, grouping of visual effects did not impact on the size of response-response binding effects. Beyvers and colleagues (2022) assessed the impact of grouping by task instructions on complex grasping responses – they found that when features like mass distribution or position repeated from prime to probe grasping movements were more accurate and stable. These findings broaden the scope of BRAC to grasping responses.

A number of studies assessed the impact of task goals or task sets on binding and retrieval. Chao and colleagues (2022) showed that task goals can influence which features are bound into event files and are later retrieved. These authors varied the task (between experiments) in the target-target paradigm of inhibition of return. They showed that a nonspatial (i.e. shape) feature is only bound and/or retrieved in a discrimination task that focusses on shape as the relevant feature, but not in tasks that focus on stimulus detection or localization. Schuch and colleagues (2022) argue that N-2 task repetition costs in the task switching paradigm may not only reflect task-level inhibition, as is typically assumed, but may also reflect episodic interference due to the retrieval of the n-2 task episodes. The re-analysis of previously published data as well as the analysis of a new pre-registered experiment provided tentative evidence for their assumption. Additional evidence for context-specific modulations of binding and retrieval processes in task-switching comes from Benini and colleagues (2022). They showed that irrelevant features of the task cues (i.e., the context) were bound together with the current task and the executed correct response. They suggest that repeating the modality (visual/auditory) or language (participants’ L2/L3) of an irrelevant cue in the subsequent trial may trigger retrieval of the bound episode which was reflected in larger response repetition benefits for task repetitions when the irrelevant cue feature repeated vs. switched. In the same vein, Qiu et al. (2022) showed completely irrelevant context features can be integrated into event files and the inter-trial variability of the context features determines the kind of binding that emerges: either the context is directly bound with the response or it is configurally bound with both the response and distractor. They used an auditory negative priming task and manipulated whether the variability of task-irrelevant context information was high across trials (eight levels of sound frequency) or low (two levels of sound frequency). Context information was directly bound to a response in the high-variability condition whereas it only modulated the binding between distractor and response in the low-variability condition.

The next block of articles extends binding and retrieval to features of time, errors and control states. Han and Proctor (2022) refer to prominent findings of foreperiod repetition effects and demonstrate that nominally irrelevant variations of foreperiods (i.e., time interval between a warning signal and an imperative stimulus) are included in event files. The authors used Bayesian modeling on trial-level data in four experiments to investigate underlying processes (including feature binding and retrieval) that contribute to sequential foreperiod effects. Likewise, Mocke and colleagues (2022) highlight that spatial as well as temporal aspects of planned actions are bound. In four online experiments, they revealed evidence that binding and retrieval are not restricted to the spatial features “left” and “right” (for which there is ample evidence), but that the features “top” and “bottom” and non-spatial features “short” and “long” are also integrated in event files. Finally, Pfister and colleagues (2022) extend this line of empirical evidence to binding and retrieval of response durations for continuous movements, yet the effects appear subtle. Limits of binding and retrieval for continuous, task-irrelevant response features are revealed by Varga et al. (2022). The authors investigated pinch responses that comprise several task-irrelevant continuous response features such as duration and force. Evidence of binding and retrieval of these features was very weak. Only response duration appeared to be integrated into event files.

Interestingly, Parmar and colleagues (2022) observed that erroneous responses are not retrieved but that the correct response (intended action goal) is included in the event file. This observation can be accounted for by the results of Foerster and colleagues (2022), who demonstrated the control states induced by previous errors are bound and retrieved by stimuli. Retrieval of cognitive control states after commission errors may thus tune future actions towards success – possibly by including a representation of the intended action goal.

The final block of articles addresses the issue of learning in terms of accumulated binding and retrieval of episodes. Huycke and colleagues (2022) provide a computational simulation on synaptic learning and synchronization (i.e., binding in the sense of the BRAC framework) to elaborate on neural foundations of accumulation of event files. Four models that differed in their ability to learn and synchronize were trained on three different action control paradigms (extensive learning; Stroop task; and Wisconsin Card Sorting task). Model comparison showed that for behavioral improvement, learning (but not synchronization) proved essential, although selective performance benefits due to synchronization appeared to depend on task requirements (e.g., difficulty). Rothermund and colleagues (2022) used the account of binding and retrieval of event files to explain proportion congruency effects in the Stroop task for confounded designs. They argue that for confounded designs with stimulus and response repetitions in subsequent trials, the proportion congruency effect (that is the finding that congruency effects are larger in blocks with frequent congruent rather than frequent incongruent trials) is eliminated when controlling for episodic retrieval of control states (the recent congruency level) and episodic retrieval of previous responses (with the latter outperforming the former in a direct comparison). Whitehead and colleagues (2022) extended findings of control bindings for longer time intervals by demonstrating that one-shot bindings of control states can last for up to 5 minutes when no interfering events are presented; yet, with interference they last with a maximum of only 2 minutes. These findings suggest that interference rather than temporal decay is the driving factor for the durability of one-shot control bindings. The time-scale of bindings is more closely considered by Dames and colleagues (2022) by assessing short-term and long-term bindings in a large-scale re-analysis of eight experiments. Results suggest that both types of bindings are dissociable because the impact of short-term bindings on retrieval of long-term bindings was feature-specific and did not generalize to broader classification categories. In addition, Fournier and colleagues (2022) showed that long-term practice effects modulated short-term bindings. In their study, practice of the S-R mappings and task reduced (but did not eliminate) partial repetition costs. Resolving confusion between action plans during retrieval is discussed within a broad framework of memory models that assume action events become more specific and less reliant on working memory with practice. Finally, Arunkumar and colleagues (2022) investigate the role of transient bindings in selective contingency learning. They observed that learning is facilitated for salient rather than non-salient stimuli, yet this result is explained by awareness rather than by stimulus-based episodic retrieval.

## Concluding remarks

Our aim was to compile a special collection full of intriguing and methodically strong papers that are related to the core processes of the BRAC framework. BRAC as an overarching framework summarizes and harmonizes research on action control – yet, as this special collection showed, there are still many details that need to be flashed out. We believe that this collection of articles is helpful to better understand factors like grouping or task goals that impact on binding and retrieval processes as well as the type of features (e.g. time, errors, or control states) that are more or less easily integrated in event files. In addition, this special collection tackled the relation between binding and retrieval and learning. In fact, binding and retrieval processes can be considered fundamental processes that build the foundation of long-lasting, learning-dependent effects for action control. Based on these contributions, this special collection fosters our understanding of basics of action control and accumulation of events as a core of procedural learning mechanisms. It also stresses the importance to separate binding from retrieval processes and treat them as building blocks of action control that can be modulated independently – the key assumption of BRAC.
